# The surgical effect on overactive bladder symptoms in women with pelvic organ prolapse

**DOI:** 10.1038/s41598-021-99537-w

**Published:** 2021-10-12

**Authors:** Ling-Ying Wu, Kuan-Hui Huang, Tsai-Hwa Yang, Hui-Shan Huang, Tzu-Shu Wang, Kuo-Chung Lan, Fei-Chi Chuang

**Affiliations:** 1 Department of Obstetrics and Gynecology, Kaohsiung Chang Gung Memorial Hospital and Chang Gung University College of Medicine, No. 123, Dapi Road, Niaosong District, Kaohsiung, 83301 Taiwan; 2 Department of Pathology, Kaohsiung Chang Gung Memorial Hospital and Chang Gung University College of Medicine, No. 123, Dapi Road, Niaosong District, Kaohsiung, Taiwan; 3 Center for Menopause and Reproductive Medicine Research, Kaohsiung Chang Gung Memorial Hospital and Chang Gung University College of Medicine, Kaohsiung, Taiwan

**Keywords:** Urinary incontinence, Hormones, Gonadal hormones, Urinary tract obstruction

## Abstract

This study aimed to explore the effect of pelvic reconstruction surgery on the relation of pelvic organ prolapse (POP) and overactive bladder (OAB) and the impact of preoperative vaginal oestrogen supplement on vaginal tissue. A total of 100 postmenopausal women with symptomatic POP who underwent pelvic reconstruction surgery (laparoscopic sacrocolpopexy or transvaginal mesh) were enrolled in this study. Preoperative vaginal oestrogen was prescribed in 28 cases. The evaluation tools consisted of POP-Q, urodynamic study, Overactive Bladder Symptom Score (OABSS), and urinary NGF. Vaginal maturation index and vaginal specimens for hormone receptors study were investigated during operation to evaluate the effect of topical oestrogen. Follow-up assessments were performed at 1, 3, and 6 months after surgery. Preoperatively, 58 (58%) were POP with OAB. After reconstruction surgery, the OABSS decreased significantly (6.87 ± 0.85 vs 3.77 ± 0.61, *p* < 0.001) at postoperative 6 months in the group. Remarkable increasing trends of urinary NGF levels are noted till 3 months postoperatively, then decreasing to the baseline level at 6 months postoperative follow-up. Remarkable decrease of mRNA of the androgen receptor and significant higher expression of progesterone receptor (PR) were noted after use of the vaginal oestrogen cream. The severity of OAB in the POP women shows moderate degree according to OABSS. Pelvic reconstruction surgery can significantly improve the OAB symptoms. The surgery induced inflammation effect lasts for about 6 months. Short-term preoperative supplement of topical oestrogen brings alterations of the vaginal epithelium.

## Introduction

Pelvic organ prolapse (POP) occurs with the loss of the normal supportive system of the pelvic floor, resulting in the descent of the pelvic organs into the vaginal canal. Many patients with POP also have symptoms of an overactive bladder (OAB), a condition of urinary urgency with or without urgency incontinence, usually accompanied by frequency and nocturia^1,2^. The actual pathophysiology of an OAB has not been fully delineated. One study postulated that metabolic derangement, bladder outlet obstruction (BOO), and inflammation can increase the excitability of nerves to the detrusor muscle and alter the sensory and barrier functions of the urothelium^3^. POP may result in kinking or compression of the urethra and cause BOO––likely the most important mechanism by which POP induces OAB symptoms^4–6^.

OAB symptoms may resolve, persist, or exacerbate after pelvic reconstruction surgery for POP. Some studies have revealed that patients with more severe POP may be at a higher risk of persistent lower urinary tract symptoms^4,7–9^. Other studies have found that the absence of bothersome OAB symptoms or detrusor overactivity preoperatively were predictors for the absence of postoperative symptoms^10,11^.

Previous studies have indicated that urinary nerve growth factor (NGF) is a promising biomarker for a variety of lower urinary tract dysfunctions. Irritation, such as inflammation, obstruction, and denervation induce an elevation of NGF, and NGF levels are reportedly about 12-fold higher in patients with an OAB compared with normal controls^12^. In males with BOO, such as those with benign prostate hyperplasia, significantly greater urinary NGF/Cr levels were observed compared with patients without BOO; in addition, these levels decreased to normal after successful relief of OAB symptoms^13^. However, there is scant data related to female BOO.

POP is frequently occurred in the postmenopausal female population. Vaginal tissue is sensitive to steroid hormones, and the lack of sexual hormones in postmenopausal women leads to insufficient blood circulation and insufficient lubrication of the vagina. Atrophic vaginal tissue appears thin, vulnerable, and less resistant to operative dissection in prolapse surgery. Treating postmenopausal women with local oestrogen before prolapse surgery results in better tissue dissection during the operation. Previous studies have shown that oestrogen therapy may effectively alleviate symptoms of an OAB^14,15^. Vaginal oestrogen cream appears to have some impact on the expression of vaginal hormone receptors, but study results have varied^16,17^.

The study aimed to explore the effect of pelvic reconstruction surgery on the relationship between pelvic organ prolapse and overactive bladder. The secondary aims were investigating the change of urine nerve growth factor level after pelvic reconstruction surgery and the impact of preoperative vaginal oestrogen supplement on vaginal tissue.

## Methods

This was a prospective study approved by Chang Gung Memorial Hospital Research Ethics Committee (IRB#201601486A3). The study was in accordance with the Helsinki Declaration of 1975, which was revised in 1983. To ensure patients’ confidentiality, only data with the encoded identification numbers were released. Postmenopausal women with symptomatic POP who underwent pelvic reconstruction surgery from September 2017 to December 2019 were enrolled in this study. Menopause is defined as no menstruation for more than 12 months. When the patients with POP visited our out-patient department, we discussed with them about the project, pros and cons, and asked them if they are willing to join the research. Thereafter, written informed consent was obtained before survey initiation. Patients were excluded if they had any neurological disease, symptomatic urinary tract infection, un-investigated haematuria, bladder cancer, interstitial cystitis, had previous pelvic reconstruction or anti-incontinence surgery or would receive concomitant anti-incontinence surgery during reconstruction surgery, or had any condition that was a contraindication for oestrogen treatment. The contraindications of using estrogen included undiagnosed abnormal genital bleeding, known or suspected breast cancer, known or suspected estrogen-dependent neoplasia and active or history of thromboembolic disease. POP severity was graded via the Pelvic Organ Prolapse Quantification system (POP-Q)^18,19^. Patients were classified into two groups according to the presence or absence of OAB symptoms, which were defined according to the patient’s complaint of urgency and/or urge incontinence as the core symptom with total OABSS score as 3 or more. Preoperatively, patients willing to receive hormone therapy were prescribed Premarin vaginal cream 1 g (0.625 mg conjugated equine oestrogens per gram of cream) for twice per week. Reconstruction surgery would be performed after the patient used Premarin vaginal cream at least two weeks.

The preoperative assessment included baseline characteristics, POP severity, urodynamic study, overactive bladder symptom score (OABSS) and urinary NGF. OABSS is a validated questionnaire including four questions (daytime frequency, night time frequency, urgency and urge incontinence) to assess the severity of overactive bladder symptoms^20–23^. Pap smears for VMI was collected from the lateral vaginal wall after anesthesia. When performing reconstruction surgery, excessive vaginal wall would be trimmed during colporrhaphy and 0.5 × 0.5 cm in size at least of this specimen would be sent for evaluating hormone receptor expression. Postoperatively, follow-up assessments comprising the POP-Q system, OABSS, and urinary NGF were performed at 1, 3, and 6 months.

### Urine sample collection and urinary NGF measurement

A 30 ml urine sample was obtained at the outpatient clinic interview. Urine samples were immediately placed on ice and transferred to the laboratory. Freshly voided urine was centrifuged at 4 °C and at 4000 rpm for 10 min. The supernatant was separated into aliquots and frozen at – 80 °C. One of the aliquots was used to measure the urinary creatinine level.

Urinary NGF measurements were performed with an enzyme-linked immunosorbent assay (Abcam ab193760, Cambridge, UK). All reagents, samples, and standards were prepared as instructed. Exactly 50 µl of each standard or sample, and 50 µl of the antibody cocktail, was transferred into each well, and then incubated for 2.5 h at room temperature with shaking. After the plate was washed, 100 μl of a tetramethylbenzidine (TMB) substrate solution was added to the wells for 10 min at room temperature. Stop Solution was added to terminate the reactions, and the optical density was measured with a Wallac Victor 1420 multilabel HTS counter (PerkinElmer Inc., MA, USA) at 450 nm. All of the samples were run in triplicate, and the values were averaged. The total urinary NGF level was further normalized by the concentration of the urinary creatinine (Cr) (NGF/Cr level).

### Expression of hormone receptor

The total RNA was isolated using the RNA Clean & Concentrator-5 kit (R1014, ZYMO RESEARCH, CA, USA) and reverse transcribed. Real-time reverse transcription-PCR (RT-PCR) was performed using a Fast SYBR Green Master Mix (Applied Biosystems, CA, USA). Sequence analysis was performed with an ABI 7500 Fast Real-Time PCR System (Applied Biosystems, CA, USA). The list of all primers used in this study are presented in Supplementary Table 1.

### Immunohistochemistry (IHC) for hormone receptor

Tissues were paraffin-embedded and subjected to immunohistochemical staining. The sections (2.5 μm) were deparaffinized and rehydrated by rinsing in purified water and treated with 3% H_2_O_2_ for 15 min at room temperature. After rinsing three times with purified water, they were heated in an autoclave for 1 h with 10 mM citrate buffer and then incubated with primary antibodies. The estrogen receptor (ER)-α (EP1, Bio SB, CA, USA), estrogen receptor (ER)-β (14C8, Abcam, Cambridge, UK), progesterone receptor (PR) (NCL-L-PGR-312, Leica Biosystem, Benton Lane, UK), and androgen receptor (AR) (Clone SP107, ZECA, CA, USA) antibodies were diluted to a concentration of 1:50. After the appropriate secondary IgG antibody was applied, the sections were incubated with DAB (K5007, Dako, Denmark) and counterstained with haematoxylin (1.05174, Merck, MA, USA). Finally, the sections were dehydrated in a graded series of ethanol, cleared with xylene, mounted in HISTOMOUNT (008030, Life technologies, MD, USA), and evaluated by light microscopy after receiving cover slips. The proportion of stained cells and the extent of the staining were used as immunoreactive score (IRS) scale for evaluation by a blinded, independent pathologist. The percentage of positive cells was assigned a score from 0 (no positive cells), 1 (< 10% positive cells), 2 (10–50% positive cells), 3 (51–80% positive cells), or 4 (> 80%positive cells).

### Vaginal maturity index (VMI) assessment

Cytological evaluation was performed using vaginal smears collected from the vaginal lateral wall and evaluated in our pathology department. The cytotechnologist estimated the proportion of parabasal, intermediate, and superficial cells, summing to 100, in the sample. All examinations were interpreted by the same blinded cytopathologist.

### Statistical analysis

Data were analysed using Graph-Pad Prism version 5 (https://graphpad-prism.software.informer.com/5.0/) and IBM SPSS Statistics 19 (https://ibm-spss-statistics.software.informer.com/19.0/). Statistical analysis was performed using the Student’s t-test for comparison of the means. The differences in urinary NGF/Cr and OABSS at baseline, 1, 3 and 6 months postoperatively were compared using the one-way analysis of variance (ANOVA) with post hoc Scheffé test. Changes of vaginal sex hormone receptor and vaginal maturity index (VMI) were performed using the Student’s t-test or Mann–Whitney U test for comparison of the means. *P-values* < 0.05 were considered statistically significant.

## Results

**Figure 1:**
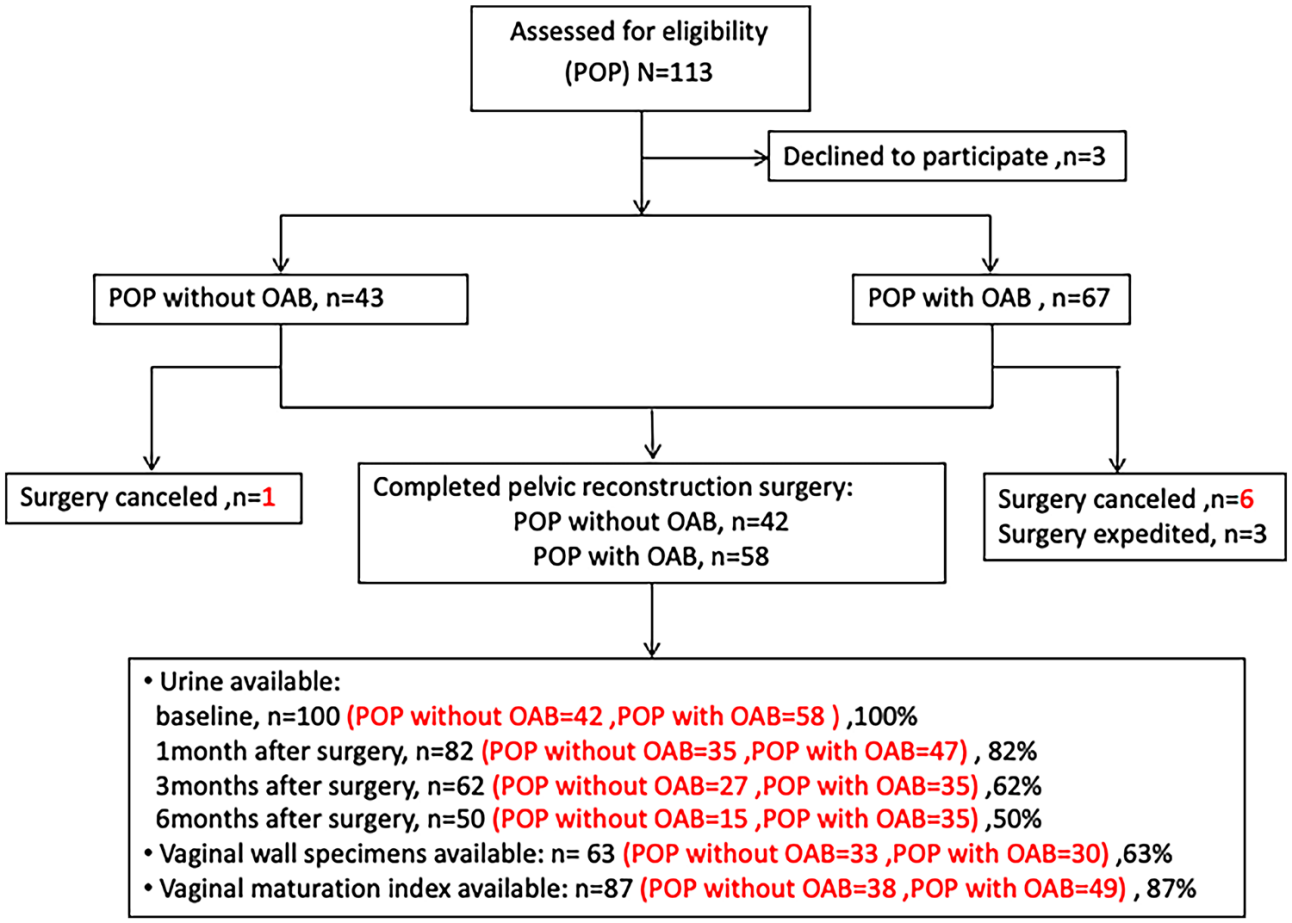
Study recruitment and follow-up.

A total of 113 patients were enrolled in this study initially. Figure 1 shows the flowchart of this study selection process. Finally, 100 patients divided into two groups as POP without OAB (n = 42) and POP with OAB (n = 58) were evaluated in this study. Patients’ demographic and characteristics are listed in Table 1. The age, severity of POP, and the ratio of surgical route are no significant differences in the two groups. The proportions of patients with POP stage II–III were higher than stage IV in both groups (65.1% vs 34.9% in POP without OAB group, 81% vs 19% in POP with OAB group). Transvaginal mesh (TVM) was performed in 85.7% (36/42) and 87.9% (51/58) in each group, others were laparoscopic sacrocolpopexy (LSC).

**Table 1:** Baseline demographic and clinical characteristics.

	POP without OAB (n = 42)	POP with OAB (n = 58)	*p*-value
Age (years)^a^	66.9 ± 6.5	67.2 ± 6.7	0.490
BMI (kg/m^2^)^a^	24.3 ± 3.9	24.9 ± 4.5	0.445
**Parity**^**b**^	3 (2–6)	3 (1–7)	
≦3	27 (64.3)	52 (89.7)	0.002
> 3	15 (35.7)	6 (11.3)	
C/S^b^	6 (14.3)	2(3.5)	0.207
Hypertension^b^	20 (47.6)	24 (41.4)	0.700
Diabetes^b^	7 (16.7)	16 (27.6)	0.242
History of hysterectomy^b^	2 (4.8)	6 (10.3)	0.310
**Stage**^**b**^			
II–III	26 (65.1)	47 (79.3)	0.167
IV	16 (34.9)	11 (19.0)	
**Route of reconstruction surgery**^**b**^			
LSC	6 (14.3)	7 (12.1)	0.589
TVM	36 (85.7)	51 (87.9)	
Preoperative Premarin use^b^	7 (16.7)	21 (36.2)	0.032

Data presented as mean ± SD, median (range), or n (percentage).

*LSC* laparoscopic sacrocolpopexy, *TVM* transvaginal mesh.

^a^Data analyzed using Student’s t-test.

^b^Data analyzed using Chi-square test.

**Figure 2:**
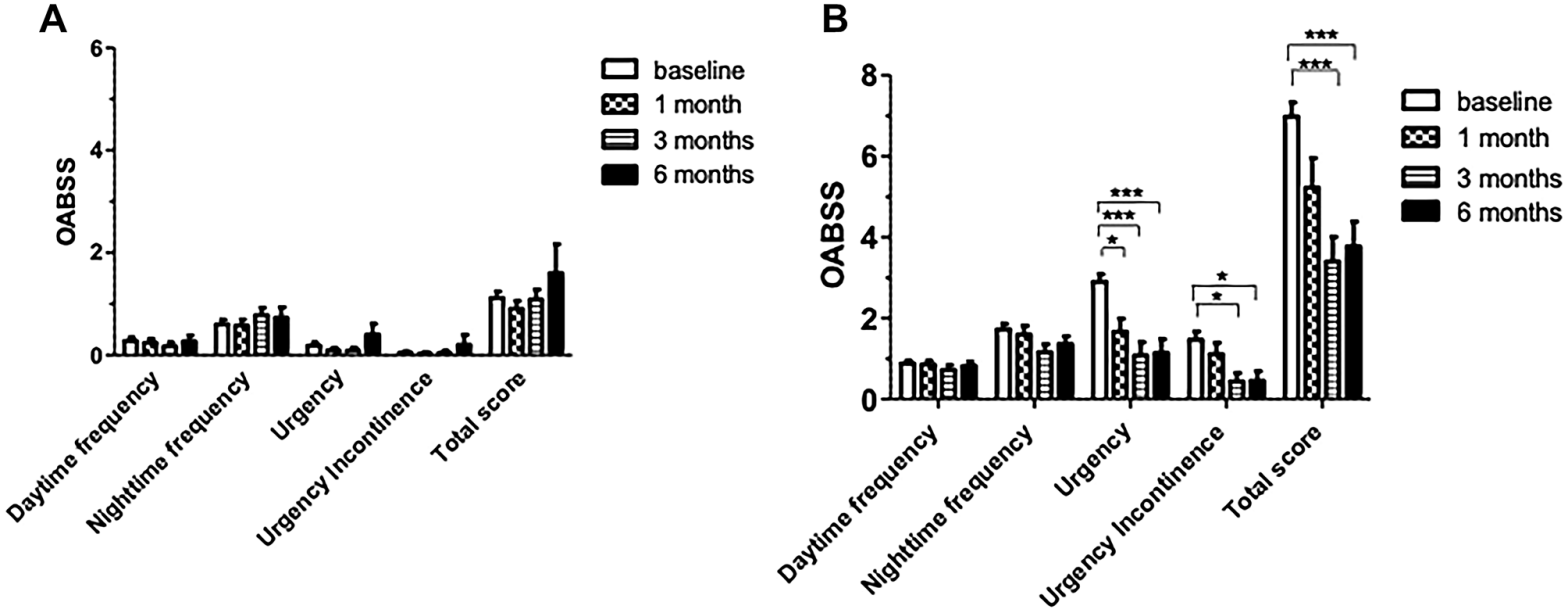
The changes of overactive bladder symptoms score (OABSS) at baseline and postoperative follow-up in (**A**) POP without OAB and (**B**) POP with OAB groups. One-way analysis of variance (ANOVA) was used to test for statistical significance between baseline, 1, 3 and 6 months postoperatively group. (Group I: baseline; Group II: 1 month; Group III: 3 months; Group IV: 6 months). Urgency: Group I vs Group II, *p* = 0.049; Group I vs Group III, *p* < 0.001; Group I vs Group IV, *p* < 0.001. Urgency Incontinence: Group I vs Group III *p* = 0.037; Group I vs Group IV, *p* = 0.046. Total score: Group I vs Group III, p < 0.001.; Group I vs Group IV, *p* < 0.001. *p < 0.05; *** *p* < 0.001. *POP* pelvic organ prolapse, *OAB* overactive bladder.

Total score of OABSS at baseline in POP without OAB and POP with OAB group were 1.12 ± 0.13 and 6.87 ± 0.35, respectively (Supplementary Table 2). Figure 2 presents the bar graph of the changes of OABSS at baseline and 1 month, 3 months and 6 months postoperative follow-up. Urgency, urgency urinary incontinence (UUI), and OABSS total were significantly improved in POP with OAB patients at 3 and 6 months, postoperatively.

Table 2 provides the values of sequential urinary NGF at baseline and postoperative follow-up in total patients, POP stage II–III, and POP stage IV. There are no significant differences in the sequential levels of urine NGF among groups. From the line graph (Supplementary Fig. S1) of these data, remarkable increasing trends of urinary NGF levels are noted till 3 months postoperatively, then decreasing to the baseline level at 6 months postoperative follow-up in all cases independent of POP stage.

**Table 2:** Levels of urinary NGF at baseline and postoperative follow-up between stage II-III and stage IV pelvic organ prolapse patients.

Stage	II–III	IV	*p*-value	Total
**NGF/Cr (pg/mg)**				
Baseline	25.38 ± 6.17	11.89 ± 3.88	0.067	21.39 ± 4.88
1 month	64.85 ± 12.87	83.06 ± 32.21	0.511	69.28 ± 12.28
3 months	101.00 ± 20.46***	111.83 ± 41.53^†^	0.812	103.66 ± 19.16
6 months	38.16 ± 14.04*	15.96 ± 10.87	0.365	32.20 ± 10.72

Item listed as mean ± SEM.

Student’s t test was used to test for statistical significance between stage II–III and IV group.

One-way analysis of variance (ANOVA) was used to test for statistical significance between baseline, 1, 3 and 6 months postoperatively group. ***Statistically significant difference in comparison with baseline (*p* < 0.001). *Statistically significant difference in comparison with 3 months (*p* = 0.026). ^†^Statistically significant difference in comparison with baseline(*p* = 0.033).

**Figure 3:**
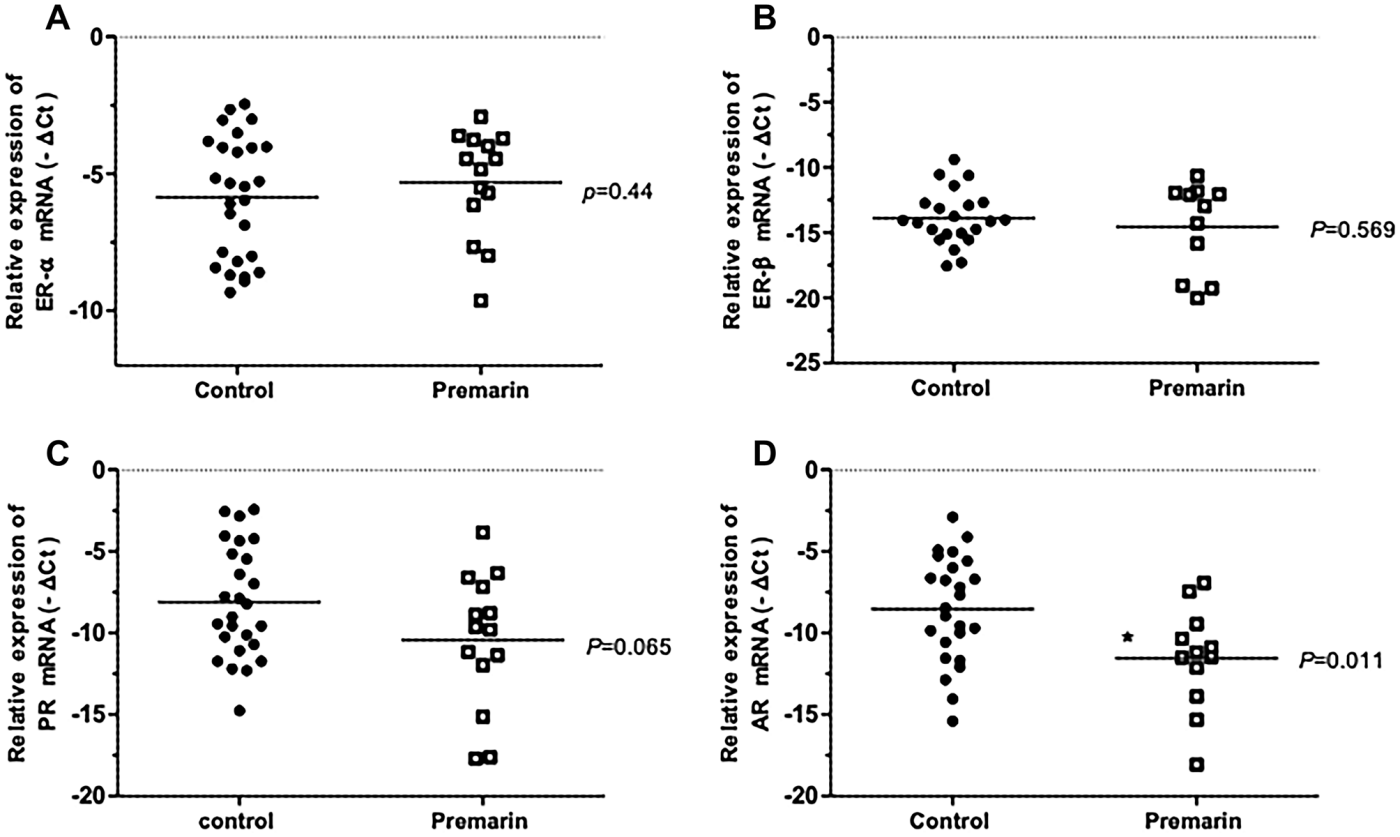
Relative expression of hormone receptors mRNA in vaginal wall biopsies of patients. (**A**) oestrogen receptor-α (**B**) oestrogen receptor-β (**C**) progesterone receptor (**D**) androgen receptor. *p < 0.05.

**Figure 4:**
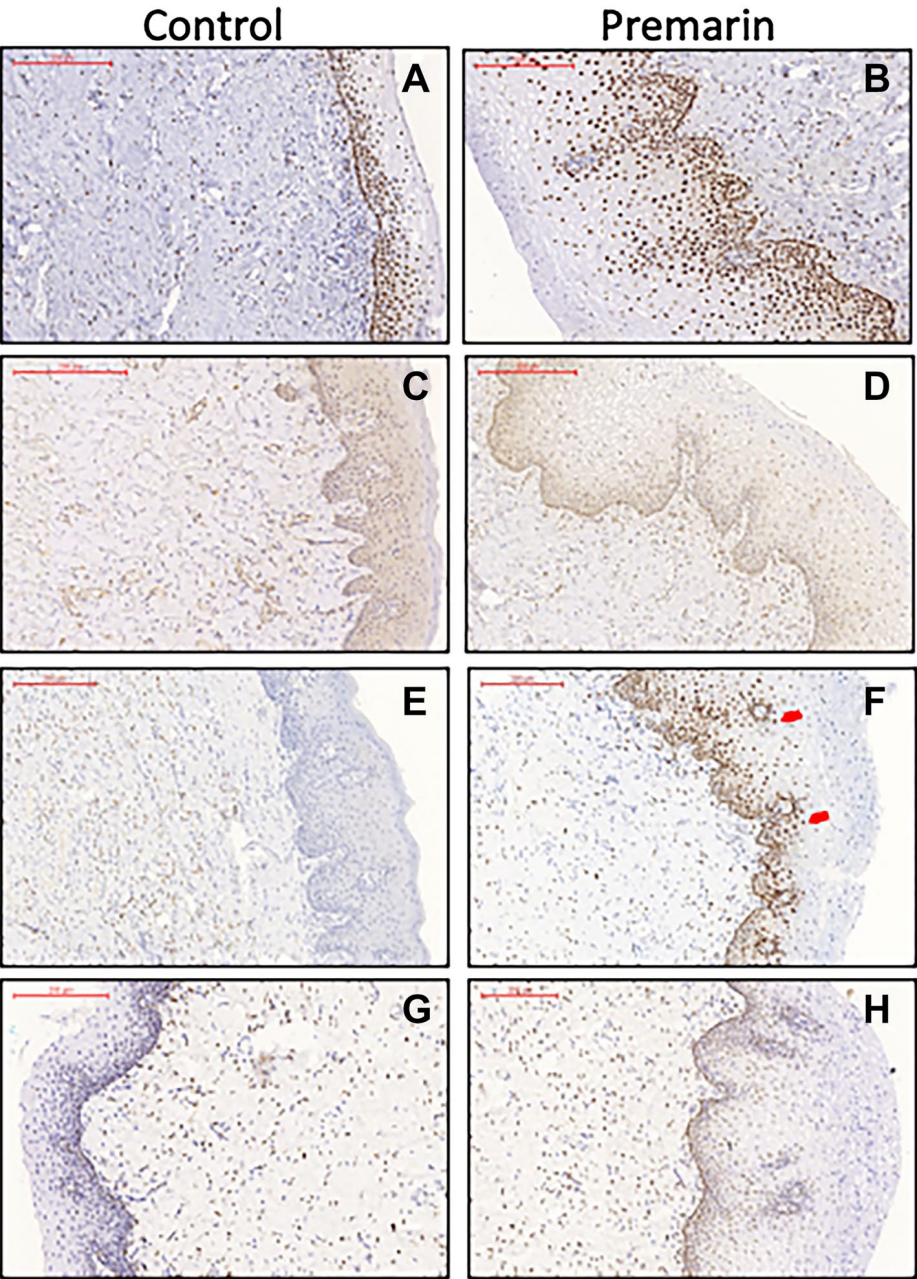

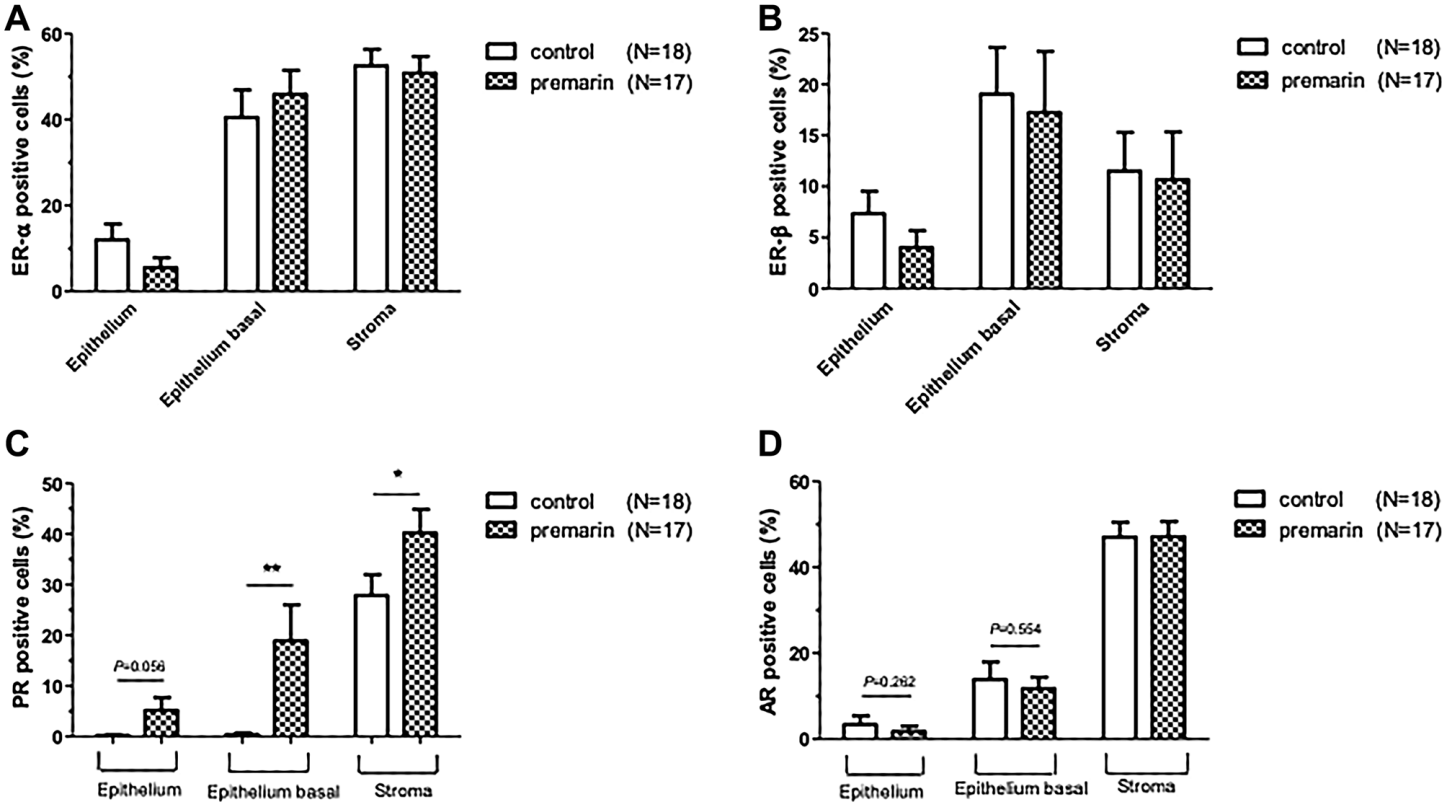
(**A**) Immunohistochemical staining of oestrogen receptor-α (A,B), oestrogen receptor-β (C,D), progesterone receptor (E,F), androgen receptor (G,H) of the vaginal wall in each group. (Control group: post-menopausal women untreated with vaginal premarin cream before surgery Premarin group: postmenopausal women used vaginal premarin cream at least 2 weeks preoperatively). Red arrows in (F) show the higher amount of PR positively stained cells (brown color) compared to the biopsy from control (E). (**B**) Immunohistochemistry quantification as percent of (A) oestrogen receptor-α (B) oestrogen receptor-β (C) progesterone receptor (D) androgen receptor positive cells in the vaginal wall between the control and intervention groups. *p < 0.05; ** *p* < 0.01.

A total of 28 patients used topical vaginal Premarin cream 1 g twice per week for at least 2 weeks before surgery. While seven (7/42, 16.7%) patients were in the group of POP without OAB, 21 (21/58, 36.2%) patients were in the group of POP with OAB. Figure 3 illustrates the relative expression of hormone receptors mRNA in vaginal wall biopsies of patients. The AR mRNA remarkably decreased after vaginal oestrogen cream supplement, whereas ER and PR retained similar expression (Fig. 3). The immunohistochemistry results are shown in the Fig. 4. From the slides of IHC staining (Fig. 4a), thicker epithelium of vaginal biopsies and significantly higher PR expression in the basal epithelium and the stroma are observed in patients who received vaginal oestrogen cream (Fig. 4b).

**Figure 5:**
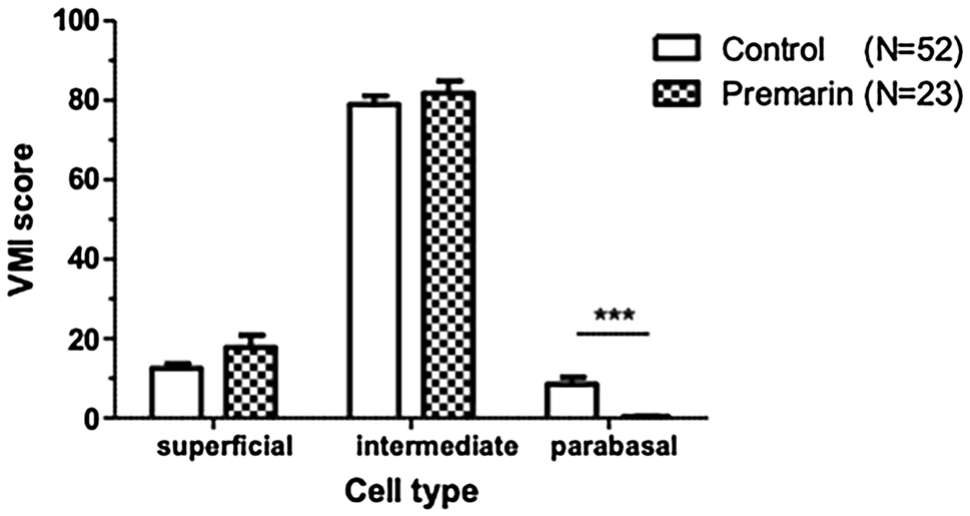
The changes in mean vaginal maturation index (VMI) score.

Vaginal oestrogen cream use changed the VMI on the parabasal cells, which significantly decreased; superficial cells were elevated without significance (Fig. 5).

## Discussion

BOO and OAB are common lower urinary tract symptoms LUTS) in POP women. A previous multicentric cross-sectional study of 521 women seeking care for pelvic floor disorders showed that LUTS are not independently associated to the prolapse severity^24^. Surgical correction of POP can relieve these related LUTS. Our study showed that 58% of POP women had OAB with a OABSS total as 6.87 ± 0.35 (moderate severity of OAB, OABSS 6–11). Urgency, UUI, and OABSS total were significantly improved at 3 and 6 months postoperatively. Among them, urgency showed immediately significant improvement at postoperative 1 month follow-up.

A prospective study, which included 34 patients who received trans-vaginal mesh for cystocele, revealed that the OABSS improved, except in the aspect of nocturia^25^. Another prospective study found 53% of POP patients had OAB before reconstruction surgery, and in 77% of them, the OAB symptoms improved or disappeared in all domains of the OABSS by the 3-month follow-up^26^. One retrospective study divided POP patients according to severity (stage 1–2 vs stage 3–4) and found that 12 months after correcting anterior or apical prolapse, urinary frequency and UUI improved without statistical difference between groups^7^.

Nerve growth factor is one of neurotrophins to promote growth and survival of sympathetic fibres and sensory nerves. In the bladder, urinary NGF is produced by urothelium and smooth muscle cells. Urinary NGF levels may be elevated in numerous conditions of pathological bladder, for instance, overactive bladder, neurogenic bladder, interstitial cystitis/painful bladder syndrome, bladder outlet obstruction, etc. A meta-analysis in 2017 reported that urine NGF/Cr cannot be used as a biomarker for OAB at present, because of lack of specificity^27^. From an evidence research of urinary biomarkers in OAB published in 2019, urinary NGF, BDNF, and ATP are increased in many OAB patients. Theses biomarkers can help identify OAB phenotypes and select the ideal patients for specific target therapies directed to neurotrophic and purinergic pathways^28^. The involvement of NGF in afferent pathway plasticity after BOO such as in benign prostatic hypertrophy has been proposed^29^. Liu and Kuo reported that urinary NGF levels increased in male patients with BOO with OAB and reduced after successful medical treatment^13^. The etiology of BOO in women differs from that of men, POP is the most common cause of female BOO. A few articles specifically reporting the relationship between POP and urinary NGF in female patients.

Chan et al. ever conducted a small case–control study (n = 10/10) to evaluate urinary NGF levels from women with anatomic BOO resulting from POP and/or previous anti-incontinence surgery. They showed that these levels were significantly higher when compared with age-matched controls. After surgical correction, the urinary NGF/Cr levels significantly decreased 1 month after intervention^30^. In this study with a larger patient population, we observed that the urinary NGF increased at the 1- and 3-month postoperative follow-ups and decreased to a level similar to baseline at 6-month after surgery. This trend of urinary NGF change after operation is identical regardless of the severity of POP. The increase in urinary NGF might be due to post-operative inflammation triggered by bladder dissection during the reconstruction surgery and the placement of trans-vaginal mesh. As time progressed to 6 months postoperatively, the inflammation subsided and urinary NGF declined. By tracing of medical records, none of the participants were taking steroids or immunomodulatory agent which might influence the study result by altering the inflammation status during the study period.

In our study, ER α and ER β did not significantly change after local oestrogen supplement for 1 month, while AR mRNA decreased, and PR protein increased. Carlo et al. reported that in postmenopausal patients, the levels of ER were similar to those found in premenopausal women, but as regards PR, the majority of the vagina was devoid of PR after menopause^31^. Chen et al. showed that ER α mRNA was detected in all vaginal walls of premenopausal and postmenopausal women, ER β mRNA was detected in all samples of vaginal walls from premenopausal women, but in none of those from postmenopausal women^32^. A cross-sectional study reported that the women who received 14 days of vaginal estriol, in comparison with the control group, had a significant increase in ER gene expression in the vagina, while enhanced PR gene expression was found in the endometrium^16^.

Various animal studies have investigated the modulation of AR expression in physiological conditions and after sex steroid hormone administration in the vagina, whereas human studies are scarce. In this study, we observed the decreasing expression of AR mRNA (Fig. 3) in intervention group while the IHC quantification of AR protein (Fig. 4b) showed only a decreasing trend without significant difference in epithelium and basal epithelium of the vaginal sections. The discrepancy results between mRNA expression and protein level may involve a number of complicated post-transcriptional mechanisms in turning mRNA into proteins that are not sufficiently well defined. The dynamic processes involved in protein synthesis and degradation are another factors to interfere the correlations between mRNA expression and protein level^33–35^. Two previous studies have demonstrated a decline in AR associated with age^36,37^. Anne Fuermetz et al. revealed that after completing 6-weeks of nightly vaginal or vulvar oestrogen cream (0.5 mg), ER α was significantly higher in the intervention group in the basal epithelium, stroma, and connective tissue, while ER β was significantly higher in the intervention group in the basal epithelium. The PR score was significantly higher in the intervention group in the superficial epithelium, stroma, and connective tissue. Local vaginal oestrogen therapy leads to an increase in ER α and PR expression of the vaginal wall in postmenopausal women, while ER β expression remains nearly unchanged^17^. As there is little evidence to support the change of hormone receptor expression after oestrogen supplementation, we could not well explain the results we found; more extensive research should be conducted in the future for better interpretation.

Regarding VMI, the predominance of parabasal cells and the absence of superficial cells indicates a low concentration of circulating oestrogens. Previous studies usually gave patients more than 12 weeks of oestrogen supplement (ex. oral synthetic conjugated oestrogens B 0.3 mg per day, transdermal patch releasing 14 μg of E2 per day, or vaginal ring releasing 7.5 μg of E2 per day), and the proportion of parabasal cells significantly decreased^38,39^. In our study, preoperatively applying vaginal Premarin cream 1 g twice per week for 2–4 weeks decreased the proportion of parabasal cells. This demonstrates that short-term low-dose local oestrogen supplement can also improve the VMI.

There are still some limitations in this study. First, all patients were postmenopausal women, there was a lack of a control group for comparison. Second, there were variations in the duration of preoperative supplement of vaginal oestrogen and it was not randomized. Third, missing data of follow-up collection may interfere the power of analysis.

In conclusion, 58% of POP women had OAB which was graded as moderate severity based on OABSS. Urgency, UUI, and OABSS total were significantly improved at 3 and 6 months postoperatively. Surgical correction of anatomical BOO provided an effective relief of OAB in POP women. Urinary NGF could not be utilized as a predictive factor of changes in OAB after reconstruction surgery because the inflammation reaction triggered by the surgery may induce significant increase in urinary NGF at 3 months postoperatively and be back to the baseline 6 months after surgery. Short-term low dose local oestrogen supplement could improve the VMI, decrease the expression of AR mRNA and increase the expression of PR protein in the vaginal tissue.

## Supplementary Information

Supplementary Information.

## Data Availability

The data used to support the findings of this study are available from the corresponding author upon reasonable request.
