# Supplementary material for: The surgical effect on overactive bladder symptoms in women with pelvic organ prolapse
Source: Sci Rep. 2021 Oct 12;11:20193. doi: 10.1038/s41598-021-99537-w (PMC8511333; doi:10.1038/s41598-021-99537-w)
Supplement: Supplementary file 1 — Supplementary Information. [file 41598_2021_99537_MOESM1_ESM.pdf]

The surgical effect on overactive bladder symptoms in women with pelvic organ prolapse

Ling-Ying Wu, MD[1*], Kuan-Hui Huang, MD[1*], Tsai-Hwa Yang, MD[1], Hui-Shan Huang, MD[2], Tzu-Shu Wang MSc[1], Kuo-Chung Lan, PhD[1,3**], Fei-Chi Chuang, MD[1**]

[1] Department of Obstetrics and Gynecology, Kaohsiung Chang Gung Memorial Hospital and Chang Gung University College of Medicine, No. 123, Dapi Road, Niaosong District, Kaohsiung City, Taiwan

[2] Department of Pathology, Kaohsiung Chang Gung Memorial Hospital and Chang Gung University College of Medicine, No. 123, Dapi Road, Niaosong District, Kaohsiung City, Taiwan

[3] Center for Menopause and Reproductive Medicine Research, Kaohsiung Chang Gung Memorial Hospital and Chang Gung University College of Medicine, Kaohsiung, Taiwan

* Equal contribution made by Ling-Ying Wu and Kuan-Hui Huang

** Equal contribution made by Fei-Chi Chuang and Kuo-Chung Lan

Corresponding Authors:

1. Fei-Chi Chuang

E-mail: fcc@cgmh.org.tw

Address: Department of Obstetrics and Gynecology, Kaohsiung Chang Gung Memorial Hospital and Chang Gung University College of Medicine, No. 123, Dapi Road, Niaosong District, Kaohsiung City 83301, Taiwan

Telephone: +886-7-7317123

Fax: +886-7-7322915

2. Kuo-Chung Lan

E-mail: blue@cgmh.org.tw

Address: Department of Obstetrics and Gynecology, Kaohsiung Chang Gung Memorial Hospital and Chang Gung University College of Medicine, No. 123, Dapi Road, Niaosong District, Kaohsiung City 83301, Taiwan

Telephone: +886-7-7317123

Fax: +886-7-7322915

Supplementary Table 1. Primer sequences of GAPDH, ER-α, ER-β, PR and AR for real-time quantitative RT-PCR

	Forward	Reverse
GAPDH	TGCACCACCAACTGCTTAGC	GGCATGGACTGTGGTCATGAG
ER-α	CAGGAACCAGGGAAAATGTG	AACCGAGATGATGTAGCCAGC
ER-β	ACTTGCTGAACGCCGTGACC	CAGATGTTCCATGCCCTTGTT
PR	TGAATCCGGCCTCAGGTAGTT	CGCGCTCTACCCTGCACTC
AR	TCACCGCACCTGATGTGTG	ACATGGTCCCTGGCAGTCTC

AR, androgen receptor; ER, oestrogen receptor; GAPDH, glyceraldehyde 3-phosphate dehydrogenase; PR, progesterone receptor

Supplementary Table 2. The OABSS at baseline and postoperative follow-up in POP without OAB and POP with OAB groups

OABSS	Follow-up	POP without OAB	POP with OAB
Question 1	Baseline	0.28 ± 0.07 (n=42)	0.87 ± 0.07 (n=55)
Daytime frequency	1month	0.24 ± 0.08 (n=33)	0.85 ± 0.12 (n=26)
	3 months	0.17 ± 0.08 (n=23)	0.72 ± 0.12 (n=25)
	6 months	0.27 ± 0.12 (n=15)	0.82 ± 0.05 (n=22)
Question 2	Baseline	0.61 ± 0.09 (n=42)	1.71 ± 0.13 (n=55)
Nighttime frequency	1month	0.58 ± 0.12 (n=33)	1.54 ± 0.22 (n=26)
	3 months	0.78 ± 0.14 (n=23)	1.16 ± 0.20 (n=25)
	6 months	0.73 ± 0.21 (n=15)	1.51 ± 0.09 (n=22)
Question 3	Baseline	0.19 ± 0.07 (n=42)	2.78 ± 0.19 (n=55)
Urgency	1month	0.09 ± 0.05 (n=33)	1.73 ± 0.33 (n=26)
	3 months	0.09 ± 0.06 (n=23)	1.08 ± 0.33 (n=25)
	6 months	0.40 ± 0.21 (n=15)	1.14 ± 0.34 (n=22)
Question 4	Baseline	0.05 ± 0.03 (n=42)	1.38 ± 0.19 (n=55)
Urgency Incontinecne	1month	0.03 ± 0.03 (n=33)	1.15 ± 0.29 (n=26)
	3 months	0.04 ± 0.04 (n=23)	0.44 ± 0.21 (n=25)
	6 months	0.2 ± 0.2 (n=15)	0.45 ± 0.24 (n=22)
Total score	Baseline	1.12 ± 0.13 (n=42)	6.87 ± 0.35 (n=55)
	1month	0.91 ± 0.15 (n=33)	5.27 ± 0.76 (n=26)
	3 months	1.09 ± 0.20 (n=23)	3.40 ± 0.61 (n=25)
	6 months	1.6 ± 0.57 (n=15)	3.77 ± 0.61 (n=22)

Supplementary Figure 1. Line graph of Table 2

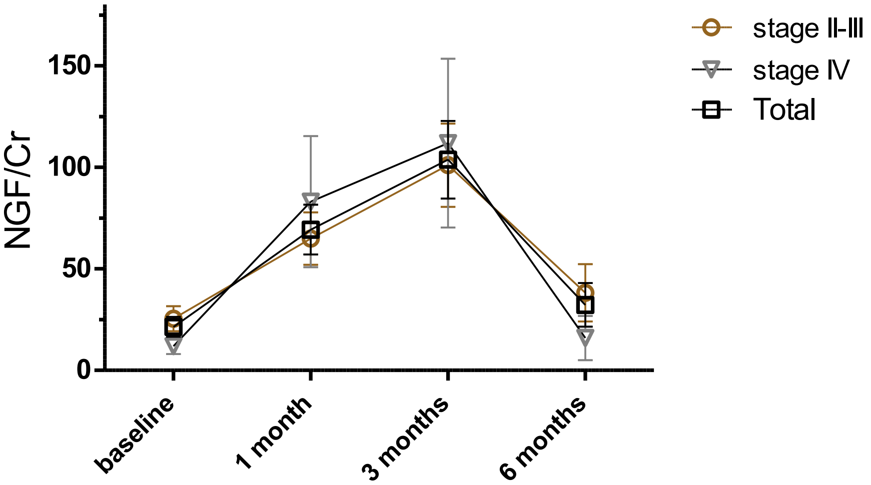

Supplementary Table 3. Urodynamic parameters before and after surgery

UD parameters	Pre-operative(n=64)	Post-operative(n=64)	*P*-value
Q_{max} (ml/s)	14.3 ± 1.2	26.3 ± 1.4	<0.001
Q_{ave} (ml/s)	4.6 ± 0.4	9.7 ± 0.6	<0.001
Voided volume (ml)	164.9 ± 16.0	341.8 ± 19.3	<0.001
Residual volume (ml)	132.2 ± 13.3	51.9 ± 7.1	<0.001
1st desire to void (ml)	138.5 ± 8.4	146.9 ± 9.0	0.341
Max capacity (ml)	329.9 ± 13.9	318.8 ± 13.0	0.364
MUCP (cmH_2O)	57.5 ± 2.5	51.9 ± 1.9	0.013
FL (cm)	2.7 ± 0.1	2.6 ± 0.1	0.248

Item listed as mean ± SEM.

Paired-t test was used to test for statistical significance between preoperative and postoperative group

Supplementary Table 4. Comparison of OAB ratio, OABSS and the urinary NGF at baseline and postoperative follow-up between control group and premarin group

	Control (n=61)	Premarin (n=24)	*P*-value
OAB n(%) [a]	30 (49.2)	18 (75.0)	0.031
OABSS[b]			
Baseline	3.7 ± 0.4 (n=61)	6.7 ± 0.8 (n=24)	<0.001
1month	2.4 ± 0.4 (n=49)	4.5 ± 0.7 (n=20)	0.013
3 months	1.5 ± 0.3 (n=44) †	3.5 ± 0.8 (n=17) †	0.028
6 months	1.5 ± 0.4 (n=38) †	2.7 ± 0.6 (n=18) †	0.113
NGF/ Cr (pg/mg) [b]			
Baseline	27.70 ± 7.20 (n=61)	10.37 ± 4.47 (n=24)	0.002
1month	82.66 ± 14.68 (n=52) †	2.50 ± 1.03 (n=18)	<0.001
3 months	136.97 ± 25.73 (n=34) †††	124.16 ± 53.73 (n=14) *,†	0.712
6 months	72.42 ± 17.49 (n=24)	25.05 ± 20.05 (n=16)	0.020

NGF: nerve growth factor

OABSS: overactive bladder symptom score

Control group: postmenopausal women untreated with vaginal premarin cream

Premarin group: postmenopausal women used vaginal premarin cream at least 2 weeks preoperatively

Item listed as mean ±SEM and n (percentage).

[a] Chi-square test was used to test for statistical significance between control and premarin group.

[b] Student's t test was used to test for statistical significance between control and premarin group.

One-way analysis of variance (ANOVA) was used to test for statistical significance between baseline, 1,3 and 6 months postoperatively group.

†, statistically significant difference in comparison with baseline(P<0.05).

*, statistically significant difference in comparison with 1 month (P<0.01).

†††, statistically significant difference in comparison with baseline(P<0.001).
